# Large-bodied squab pigeons (*Columba livia domestica*) as a genetic treasure from Central Europe

**DOI:** 10.1016/j.psj.2025.105905

**Published:** 2025-09-28

**Authors:** K. Balog, Sz. Kusza, Z. Bagi

**Affiliations:** a Centre for Agricultural Genomics and Biotechnology, University of Debrecen, 4032, Debrecen, Egyetem tér 1., Hungary; b Doctoral School of Animal Science, University of Debrecen, 4032, Debrecen, Böszörményi út 138., Hungary

**Keywords:** *Columba livia domestica*, Central Europe, Genetic diversity, Microsatellite markers, mtDNA

## Abstract

The domestic pigeon (*Columba livia domestica*) has historically contributed to food production, hobby breeding and scientific research; nonetheless, comprehensive data on its genetic diversity remain scarce, particularly for Central European breeds. This study filled this gap and applied a multi-marker approach to assess the genetic diversity and structure of seven large-bodied domestic pigeon breeds originating from North-America (*n* = 1), the Mediterranean (*n* = 2), and the Carpathian Basin (*n* = 4), using 13 nuclear microsatellite markers in 218 individuals and the mitochondrial cytochrome oxidase 1 (**COI**) region in a subset of 51 individuals drawn from the same microsatellite-genotyped samples. This design allowed for a robust comparison of biparental and maternal inheritance patterns. The combination of maternally and biparentally inherited markers provided complementary insights into genetic diversity and population relationships. Mitochondrial haplotype diversity was highest in the Runt (H_d_ = 0.964) and Hungarian Giant (H_d_ = 0.905) pigeons, and lowest in the Hungarian Cropper (H_d_ = 0.583). Microsatellite analysis revealed consistently positive inbreeding coefficients, with the highest in the Buga (F_IS_ = 0.502), suggesting inbreeding or a structured population. Among international breeds, Mondain showed the greatest genetic distance, especially from the Runt (Bruvo’s distance: 0.762), which is particularly interesting in addition to the fact of relatedness documented in the literature. Mitochondrial DNA (**mtDNA**) analysis identified 24 unique haplotypes, 13 exclusives to Hungarian breeds, reflecting the Carpathian Basin’s genetic richness. A central ancestral haplotype (Hap_17), present across all regions and in 38 individuals, suggests a common maternal origin and one domestication center. These results highlight the importance of integrating mitochondrial and microsatellite data, particularly for reconstructing breed history and identifying unique genetic resources in native pigeon populations in a specific area. Our findings, besides the scientific results, offer practical insights into the squab industry, where native breeds with rich genetic resources could support niche market expansion or the development of new, high-performance lines. The results emphasize the value of local breeds in sustainable livestock breeding. These new data create a foundation for targeted selection and conservation strategies tailored to the needs of modern pigeon production.

## Introduction

Pigeon breeding has a history of over 8,000 years, likely involving the first domesticated bird species (Johnston and Janiga, 1995; Levi, 1996). The domestic pigeon (*Columba livia domestica*) has fulfilled diverse roles from religious symbolism to meat production, yet its genetic background remains largely uncharacterized. The decline in livestock biodiversity due to intensive use of high-performance hybrids threatens all domestic species, including large-bodied squab pigeons. Genetic data on Hungarian breeds remain scarce, though essential for sustainable breeding and conservation, especially in commercially selected lines prone to bottlenecks (Faria and Miyaki, 2006; Kindie, 2021; Vekić et al., 2023). This issue is particularly relevant in the context of large-bodied Hungarian pigeon breeds, which may hold untapped potential as meat-type squabs. However, the limited literature suggests that these breeds might merely represent modified versions of certain international lines, rather than genetically distinct populations. Clarifying this through genetic analysis is essential, as disproving such assumptions could position these Hungarian breeds as valuable genetic resources for modernizing the squab sector and even for developing new meat-oriented pigeon breeds. Given the global decline in livestock biodiversity due to intensive selection, and China’s dominance in pigeon meat production through large-scale breeding, preserving and characterizing local genetic diversity has both national and international significance (Jiang et al., 2019; Ji et al., 2022; Adawy et al., 2023; Sule et al., 2024; Lan et al., 2025).

Molecular markers are valuable tools for assessing genetic variability and improving breeding strategies in animals. Using both microsatellite and mitochondrial DNA (**mtDNA**) markers allows for a complementary analysis of genetic diversity and population structure, as each responds differently to evolutionary pressures. Microsatellites, due to their high mutation rates, are particularly suited for detecting recent demographic events (Buduram, 2004; Putman and Carbone, 2014), while mtDNA provides insights into deeper phylogeographic relationships (Galtier et al., 2009). Although each marker type has its limitations, e.g., mtDNA is less informative for within-population diversity, and microsatellites can be difficult to standardize, their combined application provides a more complete genetic picture (Sharma et al., 2015; He et al., 2017). Despite this, relatively few studies have applied both marker types to the same domestic pigeon individuals. A notable exception is the study by Ramadan et al. (2011), which analyzed six Egyptian breeds using microsatellites and mtDNA COI sequences. Other studies have used multiple mitochondrial markers (e.g., COI, Cyt-b, d-loop) to investigate domestic pigeon breeds (Biray et al., 2019; Sola-Ojo et al., 2025), while multi-marker approaches have been more common in endemic pigeon species (Eltanany et al., 2011; Wilkinson et al., 2012; Lyimo et al., 2014; Ceccobelli et al., 2015; Monceau et al., 2013; Ando et al., 2014; Prakas et al., 2021; Cambrone et al., 2022, 2025). In one of the most recent studies studying pigeon breeds, Biray et al. (2025) concluded from their d-loop analysis that this marker is difficult to use as an informative phylogenetic tool for obtaining valid results in Turkish pigeon breeds, primarily due to heteroplasmy and possible introgression between different breeds. These findings highlight the need for more integrated genetic studies in domestic pigeons to better understand their diversity and support breeding and conservation efforts.

The Carpathian Basin, being one of European key biodiversity hotspots, plays a crucial role in the distribution of both wild species and domestic breeds due to its diverse ecological and geographical features (Mráz et al., 2016; Csathó and Bozó, 2022; Halada et al., 2024). As a natural arena for both genetic isolation and admixture and a historical crossroads of trade routes, the region has significantly influenced the development and diversity of domestic animal breeds, including pigeons. Its unique geographic and cultural context makes it particularly well-suited for population genetic studies aimed at exploring domestication history, especially considering that historical trade, socio-economic dynamics, climate adaptation, and breeding strategies have all shaped the genetic diversity of indigenous and local breeds (Hoffmann, 2013; Assan, 2022). Although molecular genetic studies of domestic pigeons have been carried out in parts of the Middle East (e.g., Egypt, Iraq, Turkey), various European countries (e.g., Poland, Italy, Hungary), and even in Asia and America (Ramadan et al., 2011, 2018; Biray et al., 2019; Bigi et al., 2016; Balog et al., 2024; Stringham et al., 2012), a comprehensive multi-marker analysis, especially focusing on large-bodied pigeon breeds, has not yet been conducted in Europe.

To this study, breeds were classified as “giant” if their average adult body weight exceeded 700 grams. The selection was based on official breed standards, and “giant” breeds were defined as those significantly larger than the typical pigeon (such as the racing pigeon) which generally weighs 400-500 grams and measures approximately 28-38 centimeters in length. The breeds included under this criterion are listed in Table 1.

**Table 1:** Description of the domestic pigeon breeds examined in the study.

							
**Pigeon breeds**	**King**	**Buga pigeon**	**Hungarian Cropper pigeon**	**Hungarian Giant pigeon**	**Mondain**	**Runt pigeon**	**Salonta Giant pigeon**
**Origin**	Derived from: Duchess x Homer x Maltese x Runt pigeon Country: USA XIX. Century	Derived from: the descendants of the Hungarian Giant pigeon x highflyer pigeons of Turkish origin bred Country: Hungary XVII. Century	The ancestors of the breed came to Hungary in the 1500s during the Turkish occupation Country: Hungary XVII. Century	No certain data on the origin of the breed, Similar breed is not known either on the Balkan Peninsula or in present-day Turkey. Country: Hungary XX. Century	Montauban x Runt pigeon x Bagdad ancestors Country: France XX. Century	One of the world’s oldest breeds, originating from large squab pigeons from the Campania region of southern Italy Country: France In the first century AD	The origin of the breed is not clear; it has a lot of similarity with the hungarian large-bodied pigeons Country: Hungary XX. Century
**Traits**	Straight legs, round head, fully curved body lines	Robust, stocky build, body length 40 cm Bred for resilience, and productivity	Largest wingspan among pigeons (124 cm), Sabre-shaped primary feathers, Balloon-like crop	Massive body with strong bones, well-muscled torso, prominent foot feathers, shell crest	Compact, well-muscled body with short legs, a broad, protruding chest,	Impressive size, reaching up to 55 cm in length, long, massive beak, large feet, and overall robust build	Strong skeletal structure, double crest, long, dense plumage
**Body weight**	800-960 grams	700-900 grams	850 grams	800-900 grams	1100 grams	1300 grams	900 grams

This study aimed to assess the genetic diversity, structure, and admixture of seven pigeon breeds that originate from North America, the Mediterranean region, and the Carpathian Basin, but due to their popularity being kept in many parts of the world, including Central Europe, where the individuals included in this study were sampled. To explore maternal lineages and phylogenetic relationships, allowing us to test the hypothesis that Hungarian pigeon breeds are they merely modified forms of a few international lineages. To our knowledge, this is the first European study to analyze both marker types in the same individuals, providing a more comprehensive view of domestic pigeon genetic diversity.

## Materials and methods

### Sampling and DNA isolation

Total blood samples were collected from flocks of pigeon breeders from Hungary and Romania between 2018 and 2023. The blood samples were collected in EDTA-containing tubes and stored at −20 °C until the DNA extraction.

The same individuals were processed for both marker types, but with different sample sizes, which are detailed in Table 2.

**Table 2:** Samples were used in this study.

**Breeds**	**COI mtDNA marker study sample size (n)**	**microsatellite marker study sample size (n)**	**Origin of the breed**	**Type of sample**
**Buga pigeon**	6	41	Hungary	blood
**Hungarian Cropper pigeon**	9	35	Hungary	blood
**Hungarian Giant pigeon**	7	31	Hungary	blood
**King**	8	36	United States of America	blood
**Mondain**	7	22	France	blood
**Runt pigeon**	8	30	France	blood
**Salonta Giant pigeon**	6	23	Romania	blood
**Total**	51	218		

The study was approved and conducted in accordance with the guidelines of the local ethics committee of the University of Debrecen under registration number 20/2023/DEMÁB.

DNA isolation from the blood samples was performed at the laboratory of Centre for Agricultural Genomics and Biotechnology, University of Debrecen and following the protocol by Zsolnai and Orbán (1999). The quantity and quality of isolated DNA was measured using a NanoDrop1000 spectrophotometer (ThermoFisher Scientific, USA).

### PCR reactions and capillary gel electrophoresis

#### mtDNA marker

Based on literature data, the primer pair named BirdF1 and BirdR1 in the COI region of mtDNA were selected for analysis (Awan et al., 2013). This primer pair amplified a 611 bp region that was used for the statistical analyses. DNA was amplified in a total reaction volume of 30 μl containing 7.2 µl dH_2_O, 0.6 μl of DreamTaq Flexi DNA Polymerase 1000 unit (Thermofisher Scientific, USA), 6 μl of DreamTaq Flexi Buffer, 6 µl MgCl_2_, 3 µl of dNTP, 0.6 µl (1 pmol/µl) of each primer and approximately 6 µl 50-100 ng of DNA. PCR conditions were 94 °C for 5:00 min, followed by 35 cycles of 56 °C 1:00 min, 72 °C 1:00 min, 72 °C 10:00 min. This was followed by a final extension step for 10:00 min at 72 °C and 10 °C ∞. After PCR, the success of amplification was verified by 2 % agarose gel with electrophoresis. The PCR products were sent to Macrogen Europe BV, Amsterdam for commercial sequencing.

#### Microsatellite markers

Thirteen pigeon microsatellite markers were selected from the literature (Lee et al., 2007, Traxler et al., 2000), see in Table S1. The PCR was prepared according to two different protocols with different annealing temperatures (55 °C or 60 °C), following either Protocol "A" or Protocol "B" during optimization (Table 3).

**Table 3:** Composition of PCR mix (the given amounts were used for a single sample in a 10 µl reaction mixture).

**PCR mix**	**Protocol "A"**	**Protocol "B"**
dNTP	1 µl	1 µl
5X GoTaq buffer	2 µl	1 µl
MgCl₂ (25 mM)	2 µl	0.7 µl
forward primer	0.1 µl	0.1 µl
reverse primer	0.1 µl	0.1 µl
GoTaq enzyme	0.1 µl	0.04 µl
dH₂O	3.7 µl	5.1 µl
DNA	2 µl	2 µl

The PCR conditions for Protocol „A” were 95 °C 10:00 min, 94 °C 1:00 min, 60 °C 1:30 min, 72 °C 1:00 min, 72 °C 10:00 min, 10 °C ∞. In case of Protocol „B” were 94 °C° 4:00 min, 94°C 0:30 min, 55 °C 0:30 min, 72 °C 0:45 min, 72 °C 10:00 min, then 10°C ∞.

#### Capillary gel electrophoresis

The Qiagen capillary gel electrophoresis instrument (QIAxcel Advanced, Germany) was used for gel electrophoresis. Multiplexes were created by matching primers with different fragment lengths to save material and time. The plates also contained 1 µl of size marker (QX DNA Size Marker 25 bp–500 bp) downstream of the samples. Results were evaluated using the QIAxcel ScreenGel software.

### Data analysis

#### mtDNA data

Raw sequence data was examined using Chromas software 2.6.6. version (Technelysium Pty Ltd), then the electropherograms were checked by eye and the sequences were aligned using ClustalW (Larkin et al., 2007). DnaSP 6 software (Rozas et al., 2017) was used to calculate the number and distribution of haplotypes and the nucleotide frequency values. Arlequin 3.5.2.2 software (Excoffier and Lischer, 2010) was used to perform fixation index (**F_st_**), the number of polymorphisms, genetic distance and Analysis of molecular variance (**AMOVA**) tests. The heatmaps of pairwise comparisons and visualization of the mismatch distribution data were generated from the Arlequin result output files using R 2.11.1. software. To shorten the sequences and draw the Median-joining dendrogram, MEGA11 (Tamura et al., 2021) software was used.

The phylogenetic tree was constructed using Neighbor-Joining analysis to illustrate evolutionary relationships with Population Analysis with Reticulate Trees (**Popart**) software (Leigh and Bryant, 2015), the aligned sequences were 528 bp length. To obtain a more accurate inference of phylogenetic analyses, 50 sequences downloaded from GenBank were also added to our datasets (Table S2). Common wood pigeon (*Columba palombus*) was used as an outgroup in the analysis of haplotype relationships with the Neighbor-Joining method (accession number on NCBI: MF381976.1). The haplotypes detected in this study are available in the European Nucleotide Archive (ENA) (https://www.ebi.ac.uk/ena/browser/home) genebank database under the accession number: GCA_965637565, GCA_965637575, GCA_965637585, GCA_965637595, GCA_965637605, GCA_965637615, GCA_965637625, GCA_965637635, GCA_965637645, GCA_965637655, GCA_965637665, GCA_965637675, GCA_965637685.

#### Microsatellite data

Measurement of genetic diversity parameters, such as allele frequencies, the mean number of different alleles (**N_a_**) number of private alleles, observed (**H_O_**) and expected heterozygosity (**H_E_**) and the Inbreeding coefficient (**F_IS_**) in the seven populations were estimated using the GenAIEX v.6.5. software (Peakall and Smouse, 2012). Pairwise and global F statistics (**F_ST_, F_IT_, F_IS_**), gene flow (**Nm**) and Polymorphic information content (**PIC**) for all loci and loci per breeds were calculated with R version 4.4.3. using *ape, poppr, adegenet* and *hierfstat* packages.

AMOVA analysis for each breed was estimated using Arlequin 3.5.2.2 software (Excoffier and Lischer, 2010) visualized with R version 2.11.1. The Bruvo’s distance matrix (Bruvo et al., 2004) was computed at population level using output files exported from GenAlex, for which the dendrogram and the resulting heat map were visualized using the *poppr, dendextend* and *ggplot* packages.

Taking into account the number of missing peaks, to evaluate patterns of genetic structure a more robust Structure 2.3.4. analysis version (Pritchard et al., 2010) was used to compute the dataset: Markov Chain Monte Carlo (**MCMC)** algorithm in 500.000 repetitions, 100.000 burn-in steps and 10 iterations per different K value (*K* = number of groups) (K2 – K15). The results of the analysis and the best deltaK value were validated using the STRUCTURE Harvester (Earl et al., 2012) python script, with the Evanno method (Evanno et al., 2005).

## Results

### Genetic diversity and allelic patterns revealed by mitochondrial and microsatellite markers

The genetic diversity indices reveal varying levels of genetic variation across the different values (Table 4). The Hungarian Giant pigeon breed had the highest number of polymorphisms (17) followed by Mondain (16) and the Salonta Giant pigeon (10). The lowest number of polymorphisms were found in the Hungarian Cropper breed (3) and in case of the King (7) and Runt pigeon (9).

**Table 4:** Diversity indices based on the breeds analyzed with COI mtDNA marker.

**Indices** **Groups**	**Number of elements (n)**	**Number of polymorphisms**	**Number of haplotypes**	**Haplotype diversity (H_d_) ± SD**	**Nucleotide diversity (π) ± SD**
**Buga pigeon**	6	9	4	0.800 ± 0.172	0.006 ± 0.002
**Hungarian Cropper pigeon**	9	3	4	0.583 ± 0.183	0.002 ± 0.001
**Hungarian Giant pigeon**	7	17	5	0.905 ± 0.103	0.014 ± 0.002
**King**	8	7	5	0.786 ± 0.151	0.003 ± 0.001
**Mondain**	7	16	4	0.857 ± 0.102	0.014 ± 0.003
**Runt pigeon**	8	9	7	0.964 ± 0.077	0.004 ± 0.001
**Salonta Giant pigeon**	6	10	3	0.600 ± 0.046	0.006 ± 0.003

The number of haplotypes is more distributed between the breeds. The Runt pigeon has the most haplotypes (7), while the King and the Hungarian Giant pigeon have the same number of haplotypes (5). Haplotype diversity (H_d_) follows a similar trend to the number of polymorphisms. The Runt pigeon (H_d_ = 0.964 ± 0.077) and Hungarian Giant pigeon (H_d_ = 0.905 ± 0.103) have the highest values of haplotype diversity, and the Mondain (H_d_ = 0.857 ± 0.102) and Buga pigeon (H_d_ = 0.800 ± 0.172) also showed a high value. The lowest haplotype diversity values were found in the Hungarian Cropper pigeon (H_d_ = 0.583 ± 0.183).

Nucleotide diversity was generally low, except for Mondain (π = 0.014 ± 0.003), and Hungarian Giant pigeon (π = 0.014 ± 0.002).

Microsatellite-based diversity indices are summarized in Table 5 suggesting comparable within-breed genetic diversity across the studied populations. High values of observed heterozygosity (H_O_) were detected across all studied breeds. The lowest value was detected in the Salonta Giant pigeon (H_O_ = 0.395), which does not differ substantially from the values observed in the other breeds. The highest value was detected the Hungarian Giant pigeon (H_O_ = 0.489); however, this difference is not considered notable.

**Table 5:** Diversity indices based on the breeds analyzed with microsatellite markers.

**Breeds**	**Observed Heterozygosity (H_O_)**	**Expected Heterozygosity (H_E_)**	**Fixation Index (F_IS_)**
**Buga pigeon**	0.425	0.843	0.502
**Hungarian Cropper pigeon**	0.489	0.853	0.434
**Hungarian Giant pigeon**	0.468	0.771	0.426
**King**	0.406	0.776	0.490
**Mondain**	0.417	0.781	0.482
**Runt pigeon**	0.476	0.830	0.427
**Salonta Giant pigeon**	0.395	0.778	0.499

The expected heterozygosity (H_E_) values were also found to be balanced, with the lowest value observed in the Hungarian Giant pigeon (H_E_ = 0.771), and the highest in the Hungarian Cropper pigeon (H_E_ = 0.853).

Inbreeding coefficient (F_IS_) values were likewise balanced and relatively high among the studied breeds. The highest value was detected in the Buga pigeon (F_IS_ = 0.502), while the lowest was recorded in the Hungarian Giant pigeon (F_IS_ = 0.426).

Because of the high values of the coefficient of inbreeding (F_IS_), the values per locus were also analyzed for the seven breeds in Table S3. The 13 microsatellite loci showned high levels of expected heterozygosity and polymorphic information content (PIC), indicating their informativeness and suitability for genetic diversity assessment. Observed heterozygosity values were consistently lower than expected, suggesting a high inbreeding level within populations (especially at locus PG5 with F_IS_ = 0.721). Low F_ST_ values across loci indicate limited genetic differentiation among populations, which is further supported by high gene flow (Nm) values, particularly at loci CliμT24 and CliµD01. These findings underscore the utility of the marker set for population structure analysis, despite signs of internal inbreeding not just in breeds level.

A locus-by-breed summary of F_IS_ values in Table S4, studied the inbreeding dynamics across the pigeon breeds. The Hungarian Giant pigeon consistently exhibits high inbreeding coefficients across most loci, especially PG5 (F_IS_ = 0.928) and UU-Cli14 (F_IS_ = 0.826), suggesting strong genetic isolation or prolonged closed breeding. The Salonta Giant pigeon also demonstrates elevated inbreeding values, but these values are lower.

However, the Mondain shows the lowest F_IS_ values overall (F_IS_ = 0.094 – 0.906), while the King (F_IS_ = 0.208 – 1.000) and Runt pigeon (F_IS_ = 0.170 – 0.811) breeds show moderate values compared to the others.

Locus PG5 stands out with uniformly high F_IS_ values across nearly all breeds, in contrast, loci such as CliμD35 and CliμT17 reveal lower values.

**Fig. 1:**
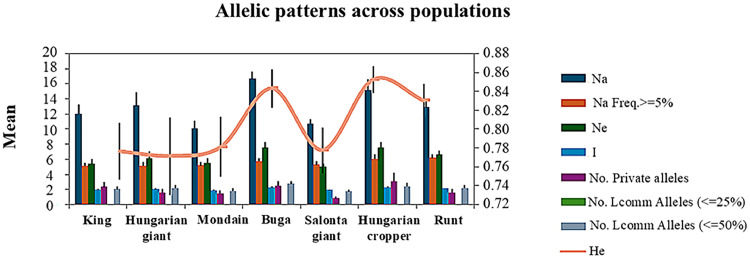
Allelic patterns across populations.

The allelic patterns across populations in Fig. 1 show that there are significant differences in the degree of allelic variation between the pigeon breeds. The Buga pigeon and Hungarian Cropper populations showed the highest allele richness (N_a_) and the number of effective alleles (N_e_), while the lowest values were observed in Salonta Giant pigeon population. High expected heterozygosity (H_e_) was observed in all studied populations.

### Genetic distance analysis from different approaches

**Fig. 2:**
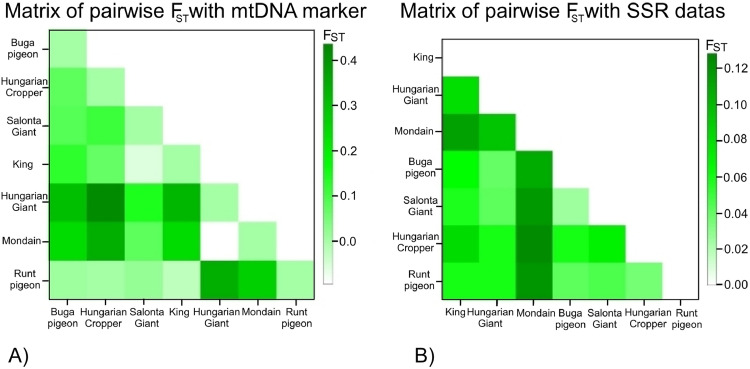
Matrix of pairwise F_ST_ with the two types of markers. The color intensity shows the higher FST values, showing the genetic distance.

Across mtDNA and microsatellite data, pairwise F_ST_ values indicate generally low inter-breed differentiation with marker-specific patterns; the full distance matrices are provided in Fig. 2A–B. Based on the analysis performed with the mitochondrial marker, no consistent pattern of clustering could be observed among the breeds (Fig. 2A), and the genetic distance values were generally low. Within this framework, the Hungarian Giant pigeon and the Mondain appeared as relatively distinct. The smallest genetic distance was detected between the Mondain and the Hungarian Giant pigeon, whereas the largest was observed between the Hungarian Giant pigeon and the Hungarian Cropper pigeon. The latter two breeds also belong to different breed groups according to the traditional, non-genetic grouping system of breeders. However, the Hungarian Giant pigeon also showed relatively high genetic distances from several other breeds, particularly the King and the Buga pigeon.

Among the international breeds, the Runt pigeon and the King appeared genetically closer to the Hungarian pigeons, while the Mondain was more distinct in both mitochondrial and microsatellite analyses. This pattern is supported by the heatmap generated from microsatellite marker analysis (Fig. 2B), where the Mondain is clearly separated from the other breeds. In this case, the Hungarian Giant pigeon showed the highest distance from the Mondain, reinforcing the trends observed with the mitochondrial data, albeit with moderate consistency.

The genetic markers used in this study vary in their levels of variability and evolutionary signal, which leads to differences in F_ST_ values and inter-population distances. These discrepancies can influence clustering patterns, or the arrangement seen in the heatmaps, as illustrated in the two panels. Indeed, the comparison of results obtained from both mitochondrial and microsatellite markers did not yield identical breed relationships, particularly for most of the studied breeds, indicating marker-specific resolution and lineage sorting.

Furthermore, the analysis using 13 microsatellite loci yielded higher F_ST_ values overall, suggesting that this type of marker provides greater resolution in detecting genetic differentiation between pigeon breeds.

**Fig. 3:**
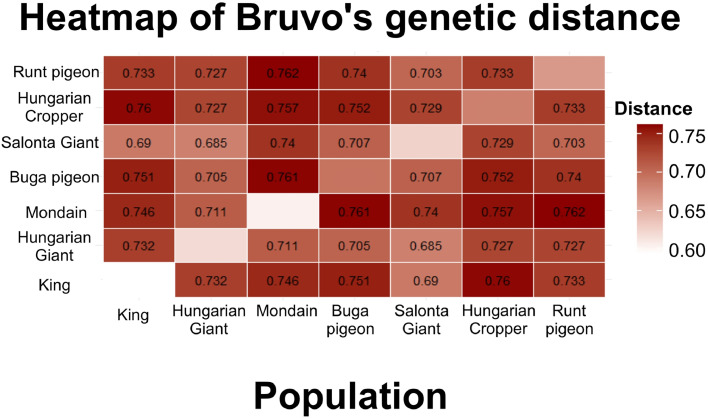
Heatmap of Bruvo's genetic distance calculated the average values for all loci in the population.

Bruvo's genetic distance considers the average values for all loci in the population, so that the previously obtained patterns can be well validated with this heatmap (Fig. 3).

A heatmap based on Bruvo’s genetic distances illustrates similar patterns detected before about the extent of genetic relationships and differentiation among the seven studied pigeon breeds. The heatmap shows that the Mondain breed is genetically distant from most other breeds, as the corresponding distance values are typically high (above 0.740), especially for the Buga pigeon (0.761) and the Hungarian Cropper pigeon (0.757). In contrast, a closer genetic relationship can be observed between the Hungarian Giant pigeon, Salonta Giant pigeon, Hungarian Cropper pigeon and Runt pigeon breeds, where the distance values are lower, for example only 0.685 between Hungarian Giant pigeon and Salonta Giant pigeon, suggesting that the breeds of the latter group might be more closely related genetically and a shared breeding history, while the Mondain breed represents a more distinct genetic lineage.

The distance values range from 0.685 to 0.762, indicating a medium level of diversity among the populations.

### Analysis of molecular variance (AMOVA)

Across marker types, AMOVA attributed more among-population variance to mtDNA than to microsatellites: 20.34 % vs. 6.84 % (Table 6). This confirms the improved applicability of the mitochondrial marker in elucidating phylogenetic questions in the domestic pigeon breeds. However, the results also show that most of the genetic diversity is maintained within individual breeds rather than between them, implying relatively low genetic structuring among the pigeon breeds.

**Table 6:** AMOVA analysis according to the breeds.

	**Source of variation**	**df**	**Sum of squares**	**Variance components**	**Percentage of variation (%)**
**COI mtDNA marker analysis**	**Among populations**	6	34.401	0.51290	20.34
	**Within populations**	44	88.403	2.00915	79.66
**microsatellite marker analysis**	**Among populations**	6	178.439	0.39413	6.84
	**Within populations**	429	2301.123	5.36392	93.16

For the microsatellite markers, the majority of the within-population variability (93.16 %) was spectacularly higher, suggesting that there is or was probably gene flow between populations, or perhaps not enough time had elapsed for divergence. Therefore, well-defined genetic clusters did not form. The predominantly within-breed distribution of variance indicates limited among-breed structure, which aligns with managed breeding and supports conservation actions that prioritize within-breed diversity.

### Demographic history inference – mismatch distribution analysis

Mismatch distributions are primarily unimodal, consistent with recent demographic expansion in most breeds, while deviations from unimodality suggest stable or bottlenecked histories in a minority of cases. So, mismatch distributions across breeds point to breed-specific demographic histories, including departures from expansion models. See Fig. S1 for the distributions by breed. In the case of Buga pigeon, the observed distribution deviates significantly from the theoretical model show with multiple peaks. This pattern indicates a highly structured population, potentially with isolated subgroups or complex demographic events such as bottlenecks or gene flow from diverse genetic sources. For the Hungarian Cropper pigeon, the observed distribution shows low pairwise differences, which only partially align with the theoretical expectations. This suggests low genetic diversity and refers to a bottleneck effect in the breed's history; this breed may originate from a restricted gene pool, likely due to selective breeding practices and the sharp decline in population size. In the Hungarian Giant pigeon, the genetic differences display a relatively broad distribution, potentially suggest substantial genetic variability or the presence of multiple genetic lineages within the breed. In the case of the King population, the observed distribution is clearly left-skewed and does not fit well with the expected distribution under demographic expansion. This pattern, along with the confidence interval values, points out a bottleneck effect and reduced genetic diversity.

In contrast, the Mondain breed again stands out among the studied populations, as the observed distribution displays multiple peaks and large pairwise differences. This may indicate high genetic diversity, possibly reflective of a population with contributions from multiple genetic sources. For the Runt pigeon, the observed distribution closely follows the theoretical model, with most pairwise differences localized within a narrow range. This suggests a stable, larger population without signs of genetic bottlenecks or other structural disturbances. In the case of Salonta Giant pigeon, the distribution is irregular and displays higher genetic differences, indicating that the breed may have undergone admixture from various genetic sources, genetic drift, or effects of isolation, which is consistent with its documented history.

### Dendrogram analyses across genetic markers

**Fig. 4:**
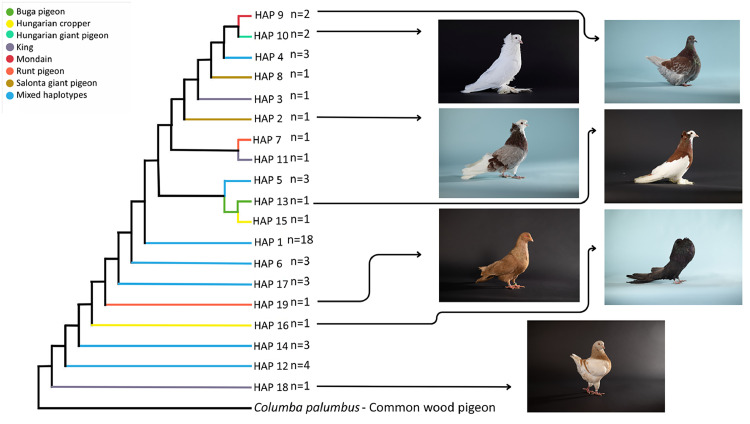
Median-Joining dendrogram of haplotype relationships based on the level of the breeds.

**Fig. 5:**
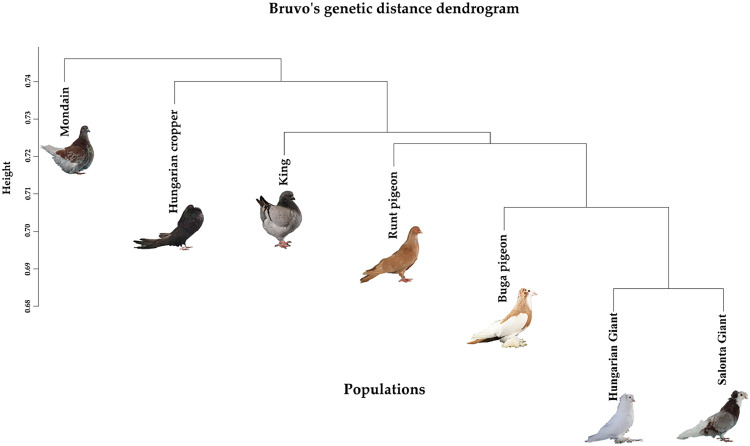
Bruvo's genetic distance dendrogram.

To visualize the genetic relationships between the studied pigeon breeds, the results of two different marker-based analyses were compared: the distribution of mitochondrial DNA haplotypes using Median-Joining analysis (Fig. 4) and the Bruvo’s genetic distance based on microsatellite markers (Fig. 5). The dendrogram based on Bruvo’s genetic distance reflects the nuclear genetic differences between breeds, the height of the clusters is proportional to the genetic distance: the higher the branch, they are more different genetically. While the tree based on the mitochondrial marker represents the genetic patterns inherited on the maternal line. Similarities between the two analyses can be observed at several points: the Salonta Giant and Hungarian Giant pigeons show a close relationship on both dendrograms, indicating that the two breeds have similar genetic backgrounds at both the nuclear and mitochondrial levels. Together, these tree-based patterns point to broadly shared ancestry with marker-specific nuances, reinforcing that both nuclear and mitochondrial signals are needed for breed-level decisions.

The Mondain breed is again at the top of the tree, in line with previous results, indicating a large genetic divergence, and is further away from the King breed in both plots. However, among the international breeds, the King and Runt pigeon are close to each other in both analysis types. Among the Hungarian breeds, the Buga pigeon is the closest to the international breeds. In the mitochondrial marker test, several breeds share common haplotypes, which may indicate crosses or common maternal ancestry. This consistent separation of Mondain across methods underscores its distinct lineage, making it a robust reference for breed identification.

Combining Median-Joining dendrograms based on COI mtDNA with Bruvo's distance dendrograms derived from microsatellite data allows for a more comprehensive understanding of both maternal lineage patterns and biparental genetic structure. The COI mtDNA tree highlights deeper phylogeographic relationships, while the Bruvo-based tree captures recent gene flow, admixture, and breed-specific diversity. Discussing them together provides cross-validation and reveals congruent or contrasting signals of evolutionary history and population structure.

### Haplotype distribution

**Fig. 6:**
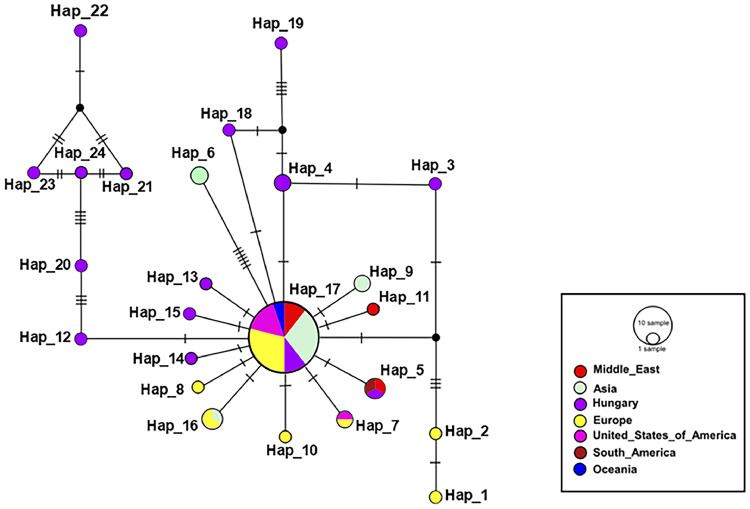
Neighbor-Joining haplotype network. The sizes of the circles indicating haplotypes are proportional to the number of individuals. Branch lengths are not always representative of evolutionary distance.

The Neighbor-Joining analysis performed using mitochondrial DNA markers is particularly effective due to the maternal inheritance, which allows for the identification of geographic distributions or separations of haplogroups and may help in tracing domestication centers (Szalay, 2017). As visualized in Fig. 6, the haplotype network provides a compact overview of these relationships. A total of 24 distinct haplotypes were identified in the network. Within these, 13 unique haplotypes are exclusively from Hungarian samples, indicating a significant genetic diversity of the Carpathian Basin population. This outstanding haplotype richness may reflect long-term historical population dynamics, mixed origins and potential isolation processes in this area. In addition to the thirteen unique Hungarian haplotypes, two haplotypes were identified that occur both in Hungarian breeds and in gene bank sequences. Of particular interest is haplotype Hap_5, which is found in one sample from Argentina, one from Saudi Arabia, and one from Hungarian breeds. Haplotype 17 has occurred in 38 individuals, mixed from all regions, representing a common, possibly ancient, origin in terms of genetic relationships. The high number of local haplotypes in Hungarian samples therefore supports the Carpathian Basin as a reservoir of unique maternal diversity with conservation relevance.

Smaller circles at the periphery represent locally distinct haplotypes, probably the result of more recent mutations, and may be signs of genetic isolation or regional adaptation. A minority of the samples from the Hungarian geographic region belong to the common haplotype, while a larger proportion is more peripheral, suggesting a potentially heterogeneous genetic background within the region.

### Cluster analysis

**Fig. 7:**
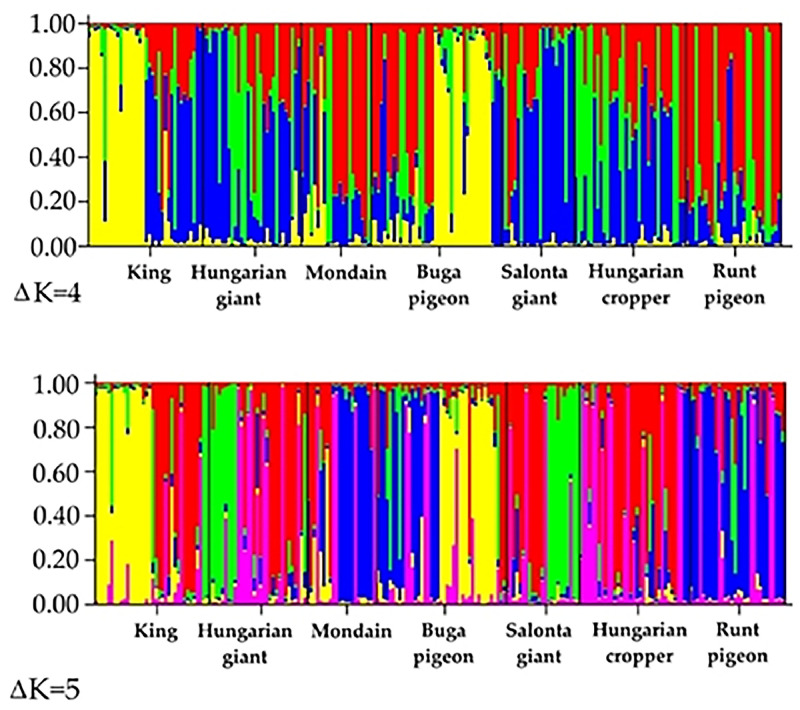
The result of the cluster analysis with STRUCTURE 2.3.4. Each column represents an individual, and the colours within each column indicate the proportion of the breeds belonging to different genetic clusters.

Bayesian clustering with STRUCTURE (admixture model, no prior population labels) supports four major ancestry components (*K* = 4) as the strongest partition based on the ΔK criterion, with a secondary peak at *K* = 5 indicating finer substructure. In practice, *K* = 4 captures the principal divisions among breeds, whereas *K* = 5 resolves additional, subtler differentiation while retaining the same broad pattern. The full assignment proportions for *K* = 4 (top) and *K* = 5 (bottom) are provided in Fig. 7, which illustrates the distribution of cluster memberships across individuals and breeds.

At *K* = 4, several breeds - including the Hungarian Giant pigeon and Hungarian Cropper pigeon -show a high degree of admixture. The distribution of colors shows that some breeds, such as King or Runt pigeon, have a more homogeneous genetic background. At *K* = 5, the resolution increases, allowing clearer distinction of population-specific genetic components. Notably, the Salonta Giant pigeon and Hungarian Giant pigeon together exhibit unique cluster dominance, suggesting bigger distinct from the studied breeds, and shown different maternal or regional genetic backgrounds. The Mondain also shows a relatively homogeneous cluster assignment, indicating genetic stability, while the Buga pigeon remains genetically heterogeneous, reflecting its transitional status and mixed origin. These results may underscore both the shared ancestry and diversification among native Hungarian and international pigeon breeds and can support the relevance of *K* = 5 as a more informative level of genetic structuring.

## Discussion

Our findings have significant international implications for the global squab-pigeon sector, particularly given the increasing commercial importance of squab production and the need for comprehensive genetic characterization of breeding resources worldwide. While many pigeon breeds exist globally, the subset of large-bodied (giant) meat-type breeds is relatively limited and historically concentrated in the Mediterranean, the United States of America and Central Europe (Carpathian Basin), where scientific documentation has been sparse. The global market context further amplifies the need for robust genetic baselines: recent sources estimate that China accounts for ≈80 % of worldwide squab production (≈680 million birds annually) and recognize pigeons as the fourth major poultry class in China (Wang et al., 2025). Moreover, squab retains cultural and culinary importance in other regions (e.g., the Middle East and parts of Europe), indicating sustained demand (Ramadan et al., 2011; Mohamed et al., 2022). In this context, characterizing and conserving the unique genetic resources of Carpathian Basin breeds while demonstrating multi-marker, cross-validated breed identification provides a transferable framework for genetic improvement programs that will increasingly require the integration of diverse gene pools to support efficient, welfare-friendly and resilient intensive production.

The genetic background of many domestic pigeon breeds remains poorly understood. This gap is especially critical in the case of large-bodied pigeon breeds, which may serve as valuable resources for sustainable meat production but there is the risk of extinction of native breeds or misclassified as variants of more popular international lines. In the context of a global decline in livestock biodiversity and increasing industrialization of pigeon farming, characterizing these local genetic resources is essential for conservation and potential breed development. By combining mitochondrial DNA and microsatellite data, this study enabled a nuanced reconstruction of microevolutionary processes in seven large-bodied pigeon breeds, with particular emphasis on those originating from the Carpathian Basin. The comparison of genetic diversity indicators revealed consistent patterns between the two marker types (microsatellites and COI mtDNA). However, distinct results emerged in certain breeds. In the case of the Buga pigeon, high inbreeding was detected (F_IS_ = 0.502) only with microsatellite markers, suggesting that using a combination of markers can help obtain more accurate results from fewer samples, as distinct markers might reveal non-overlapping patterns of population structure (Table 5) (Akib et al., 2015). The fixation indices (F_ST_, F_IS_, F_IT_) calculated for each locus showed variability, with F_ST_ values ranging from 0.017 (CliMT2) to 0.098 (UU-Cli14) (Table S3). These values may indicate meaningful population differentiation, though relatively low, especially when compared to other studies (Ramadan et al., 2011; Bigi et al., 2016). Notably, differences in marker mutation rates may result in differing F_ST_ estimates, potentially masking true population structure (Hedrick, 2005). Bruvo’s distance metric, integrating data from multiple biparentally inherited loci (Fig. 3), provided a more robust and detailed view of population relationships. All three distance methods yielded overlapping patterns, strengthening the reliability of our results. The AMOVA results (Table 6) also reinforced this finding: the COI mtDNA-based analysis showed that 20.34 % of the genetic variation was attributable to differences among breeds, while 79.66 % was found within individuals. In 2016, Bigi et al. conducted a study on Italian and Mediterranean-type pigeon breeds using a significantly larger sample size (*n* = 427). They reported a similar pattern, with 21.34 % of the total genetic variance observed among breeds and 74.69 % within individuals (Bigi et al., 2016). This pattern likely reflects the conserved nature of the COI mitochondrial DNA marker. Taken together, the modest mtDNA resolution and clearer microsatellite signal indicate recent admixture overlaid on conserved maternal lineages, a scenario typical of intensively selected domestic breeds.

In contrast, the AMOVA conducted using microsatellite markers revealed a markedly different distribution: 93.16 % of the genetic variation occurred within populations, and only 6.84 % was observed between populations. These findings align with those of Giunchi et al. (2020), who reported 90 % within-population, and 4 % among-population variation based on 12 microsatellite loci in 194 individuals – a smaller sample size compared to our study. Although AMOVA analyses using microsatellite markers are less frequently reported in pigeons, the results from both marker types in our study clearly indicate that the vast majority of genetic variance resides within populations. This highlights the exceptionally high level of genetic diversity present in domestic pigeons. This pattern can be explained by the relatively short selection history of domestic pigeon breeds; as populations have not been separated for extended periods, with modern breeds representing at most a few hundred generations of divergence. Intense crossbreeding and shared ancestry, together with breeding practices that routinely mix individuals across lines such as show breeding or market-oriented crosses, may also contribute. Among the Carpathian Basin breeds, genetic diversity patterns were highly variable. The Hungarian Cropper pigeon, one of the oldest Hungarian breeds, showed moderate genetic diversity (H_d_ = 0.583±0.183), likely reflecting geographical isolation and declining population size, which is confirmed by the information provided by the breeders. In contrast, the Hungarian Giant pigeon, showed high diversity (H_d_ = 0.905±0.103) and the lowest inbreeding coefficient (F_IS_ = 0.426), a pattern consistent across both marker types (Table 5). The Salonta Giant pigeon displayed low diversity (H_d_ = 0.600 ± 0.046) and high inbreeding (F_IS_ = 0.499), aligning with its near-extinct status and extremely small population size (Meleg, 2001). All studied breeds showed positive inbreeding coefficients, suggesting artificial selection (Ramadan et al., 2011; Biała et al., 2015; Bigi et al., 2016). Observed heterozygosity (H_O_) ranged widely from 0.208 to 0.626, these elevated H_O_ values are particularly notable when compared to previous studies on Middle Eastern pigeon breeds, where lower levels of genetic diversity were observed (Balog et al., 2024; Bayraktar et al., 2024) (Table 5). The overall high H_e_ value indicates general maintenance of genetic diversity however, the presence of private alleles and low-frequency variants suggests ongoing local genetic structuring in certain breeds. This emphasizes that management should preserve within-breed variability while monitoring limited but non-zero among-breed differentiation.

The mean F_IS_ values were higher than in comparable studies (Toalombo et al., 2019; Ramadan et al., 2018), implying stronger inbreeding or differing breeding practices. High allelic diversity (Na) further supported these trends, particularly in the Hungarian Giant pigeon and King breeds, with lowest values in the Salonta Giant pigeon and Mondain (Fig. 1). The lowest F_IS_ value in the case of Mondain (F_IS_ = 0.094) also indicates higher genetic diversity and higher gene flow, while the moderate F_IS_ values obtained in the King (F_IS_ = 0.208 – 1.000) and Runt pigeon (F_IS_ = 0.170 – 0.811) breeds may indicate inbreeding, which rather reflects on a locus-specific or subpopulation-level effects. Thus, these populations of pigeons may be highly vulnerable to loss of genetic diversity and the negative consequences that this may have. It would be worth considering genetic updating (outbreeding) or managed breeding in these populations.

Pairwise genetic distances analyzed by Arlequin were generally low, especially in COI mtDNA (Fig. 2A). The highest distances were between Hungarian Giant pigeon and Hungarian Cropper pigeon (COI mtDNA: 0.43522), and Cropper and Mondain (microsatellite: 0.12804). Despite geographic separation, Hungarian breeds retained high genetic diversity and gene flow. Two main clusters emerged: (1) King, Hungarian Giant pigeon, and Runt pigeons; and (2) Salonta Giant pigeon, Hungarian Cropper pigeon, and Buga pigeons. This was corroborated by Bruvo’s distance and heatmaps (Fig. 3). Our F_ST_ values were higher than those found in wild pigeons (Monceau et al., 2013; Giunchi et al., 2020), but comparable to European and Mediterranean type of breeds studies (Bigi et al., 2016). Compared to Polish populations (Podbielska and Radko, 2022), our breeds showed stronger differentiation and less gene flow, which may be attributed to targeted selection practices and localized breeding preferences rather than purely geographic separation. Notably, no values exceeded 0.430, implying that limited gene exchange may result more from selective breeding than from natural spatial barriers (Hall et al., 2022). Bruvo’s method proved effective in resolving these patterns due to its integrative nature. Consequently, differences between breeds at this level are modest; therefore, multi-marker approaches will be particularly useful in future breeds classification decisions, as they increase resolution and robustness by integrating different data sources, thereby reducing marker-specific biases.

Although pigeon breeding in Hungary has been organized for nearly 150 years, comprehensive quantitative data on local breeders and populations remain scarce. The most recent national survey was conducted in 2012 by Bagi. However, exhibition data provide insight into breed popularity and population trends, currently indicating that international breeds dominate over Hungarian ones. Based on the Hungarian animal husbandry is a rapidly changing sector, where population expansions and bottlenecks are frequent. Therefore, the analysis of past population dynamics using mitochondrial markers can help identify breeds with untapped or declining genetic resources (Lenstra et al., 2012). In addition to the national survey, a breed evaluation scoring system was developed, which aligns well with the findings of mismatch distribution analyses (Fig. S1) (Bagi, 2012). The Hungarian Giant pigeon is not considered endangered; its wide pairwise difference distribution reflects a genetically diverse and relatively stable population with no evidence of recent bottlenecks. In contrast, the Hungarian Cropper pigeon remains endangered, as its mismatch pattern deviates strongly from the expected model, suggesting possible inbreeding, a limited founder base (Horn, 1991), or a recent bottleneck. The Buga pigeon also shows signs of vulnerability, with multiple mismatch peaks suggesting underlying population structure. This breed, primarily from the Southern Great Plain, appears to be both genetically structured and geographically isolated (Mackrott, 2012). The most critical case is the Salonta Giant pigeon, a historically Hungarian breed now nearly extinct, surviving in small numbers in western Romania (Partium) and marginally in Hungary. Its mismatch distribution shows strong signs of isolation, with broad pairwise differences and no indication of a stable, cohesive population (Szűcs, 1990). Among international breeds, Runt showed a bell-shaped distribution suggesting expansion. Mondain had high diversity and multimodal distribution, reflecting mixed ancestry. The Mondain is commonly occurring breed in Hungary. Mondain breed was selectively developed in the early 20th century and officially recognized as a breed in 1931, making it a relatively old and established lineage (Sell, 2012). The King breed presents a skewed distribution concentrated at low differences, likely due to strong selection pressure, with moderate overall diversity. It is one of Hungary’s most popular international breeds, the first imported King pigeons arrived at Hungary in 1958 (Bagi, 2012).

Dendrograms (Fig. 5, Fig. 6) highlighted the genetic distinctiveness of the Mondain breed and supported admixture in some Hungarian breeds. Shared haplotypes between Hungarian Giant pigeon and Salonta Giant pigeon point to a common ancestry despite different demographic trajectories. Buga pigeons appeared genetically intermediate between domestic and international breeds, suggesting historical gene flow. Both the King and Runt pigeon are widely bred international large-bodied breeds. While moderately differentiated from the Hungarian breeds, they show close genetic affinity with one another, which is consistent with their breeding history – King pigeons were developed in the 1890s by crossing Duchess, Homer, Maltese, and Runt breeds. This is further supported by the haplotype tree, where both breeds cluster with distinct but adjacent haplotypes, and by their genetic proximity in the Bruvo’s dendrogram, suggesting a combination of independent genetic development and occasional introgression (Nowicki et al., 2007; Sell, 2012; Shapiro et al., 2013). The origin of the Mondain pigeon breed date back to 19th-century France, where the primary breeding goal was to develop a fast-growing squab-type pigeon. To achieve this, breeders used several different breeds in the crossbreeding process, including the Montauban pigeon, Runt pigeon, and most likely Bagdet-type pigeons (Horn, 1991; Sell, 2009). This diverse genetic foundation, combined with the fact that the breed was developed in various regions under differing selection criteria, contributed significantly to the high genetic diversity of the Mondain (Levi, 1996). The genetic diversity of the breed has also been confirmed by recent studies: the Mondain's variability is likely due to its broad genetic foundation and the fact that it has not undergone the same degree of inbreeding over the decades as many other domestic pigeon breeds (Eggleston, 1921; Charonnat, 2016; Balog et al., 2024). The central position of Hap_17 (38 individuals) suggests an ancestral haplotype, which supports the hypothesis of a single domestication center previously proposed in earlier studies (Stringham et al., 2012; Shapiro et al., 2013; Biray et al., 2019). The integration of COI mtDNA and microsatellite markers enabled a detailed reconstruction of the genetic background of domestic pigeon breeds with diverse breeding histories and geographic origins. While both markers demonstrated high overall genetic diversity-especially in some native Hungarian breeds-the results were not always concordant across different analytical approaches. In certain cases, microsatellite markers suggested higher levels of genetic admixture, whereas the COI mtDNA marker revealed stronger differentiation. These discrepancies are likely due to the inherent differences between marker types: mtDNA reflects maternally inherited, long-term lineage patterns, while microsatellites capture biparental, more recent population-level structure and gene flow. In addition to revealing marker-specific patterns, it is also important to consider the impact of sample size on genetic analysis. A previous large-scale study on Carpathian Basin pigeon breeds (Balog et al., 2024), involving 276 individuals, revealed COI mtDNA patterns closely aligned with the current findings. Despite the larger sample size, genetic boundaries between breeds were often blurred and admixture was evident in several populations, resulting in similarly low F_ST_ values. Regardless of sample size or breed classification method, the majority of genetic variance was consistently observed at the individual level - just as in our present study. Moreover, comparative analysis with two Iraqi breeds further confirmed the high level of genetic diversity within domestic pigeons and suggested that large-bodied breeds from the Carpathian Basin form a somewhat distinct cluster relative to other giant breeds. These findings also support previous conclusions that mitochondrial marker analyses are likely less sensitive to sample size fluctuations (Ramadan et al., 2011), emphasizing the importance of applying a multi-marker approach when studying genetically diverse populations. The Carpathian Basin appears as both a transitional genetic area and a reservoir of unique haplotypes, emphasizing its dual role in the preservation and connection of pigeon genetic diversity (Thomson et al., 2014; Balog et al., 2024). These consistent signals bolster our interpretation of breed identities and provide a practical framework for breed identification and targeted conservation.

At the same time, given the modest per-breed mtDNA counts (6–9; total *n* = 51) and the rarity of several breeds, the next paragraph outlines the study’s main limitations and the rationale for a multi-marker interpretation. Per-breed sample sizes are modest due to the rarity of several breeds. Accordingly, breed-level findings from these strata should be viewed as indicative rather than definitive. In our interpretation, we therefore rely on concordant evidence across markers (microsatellites for population-level structure and mtDNA for maternal context) and report patterns with appropriate caution. Notably, a previous larger-sample mtDNA analysis including of the same breeds (Balog et al., 2024) recovered relationships concordant with those reported here, supporting the robustness of the broad patterns despite modest per-breed mtDNA n. While the main signals of differentiation appear robust at the scale considered, larger future datasets will be essential to refine breed-level estimates and to test fine-scale hypotheses.

## Conclusion

Our findings show that, despite the long history of artificial selection in pigeons, overall genetic diversity remains high-especially in some native Hungarian breeds, which not only exhibit unique genetic features but also, as evidenced by the two marker analyses, challenge the notion that they are merely local variants of international lines, instead reflecting independent genetic evolution driven by geographic isolation and reduced selection pressure. The partially overlapping but occasionally divergent results obtained from the two marker systems emphasize the importance of a multi-marker strategy. Such an approach offers a more comprehensive view of genetic diversity and is particularly useful for evaluating small populations in support of conservation and breeding decisions.

## CRediT authorship contribution statement

**K. Balog:** Writing – original draft, Visualization, Validation, Software, Methodology, Investigation, Formal analysis, Data curation. **Sz. Kusza:** Writing – review & editing, Supervision, Resources, Funding acquisition. **Z. Bagi:** Writing – review & editing, Validation, Supervision, Project administration, Methodology, Conceptualization.

## Disclosures

The authors declare that they have no known competing financial interests or personal relationships that could have appeared to influence the work reported in this paper.

## Appendix Supplementary materials

Image, application 1

Image, application 2

Image, application 3

Image, application 4

Image, application 5

## Footnotes

Supplementary material associated with this article can be found, in the online version, at doi:10.1016/j.psj.2025.105905.
