# Supplementary figures and images for: Large-bodied squab pigeons (Columba livia domestica) as a genetic treasure from Central Europe
Source: Poult Sci. 2025 Sep 28;104(12):105905. doi: 10.1016/j.psj.2025.105905 (PMC12549551; doi:10.1016/j.psj.2025.105905)

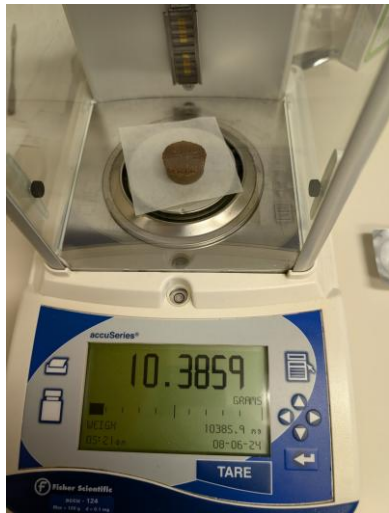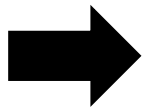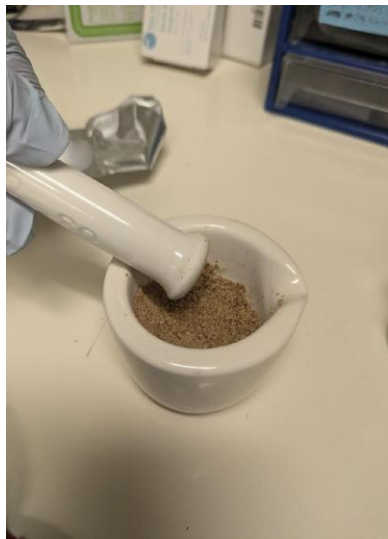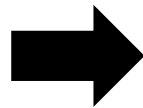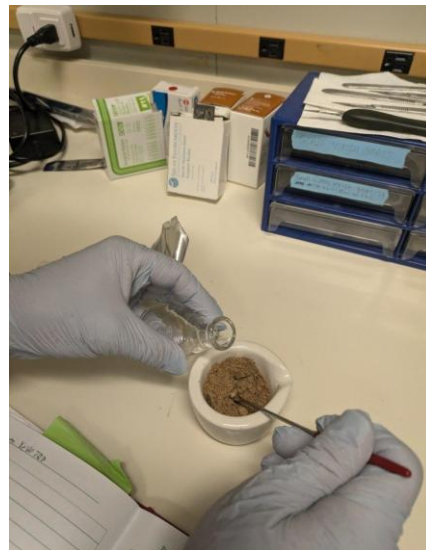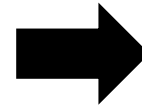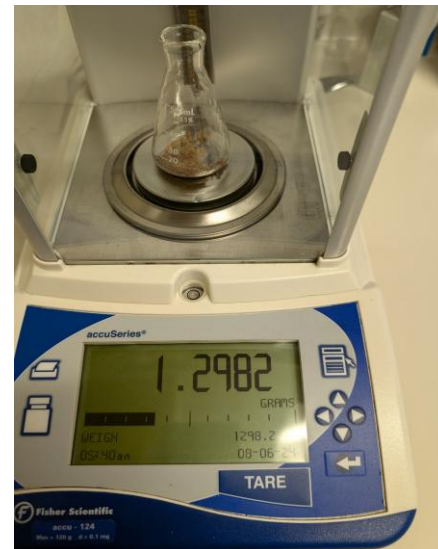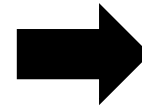

1.

2.

3.

4.

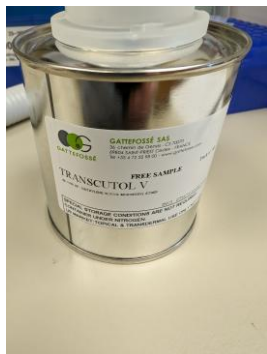

5.

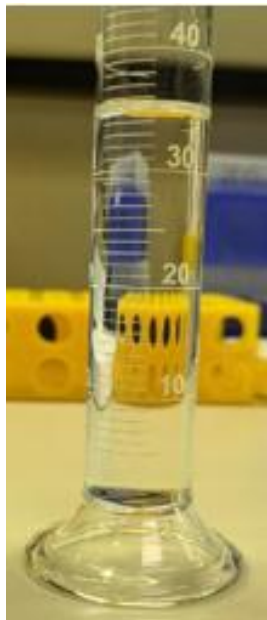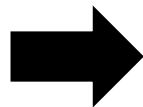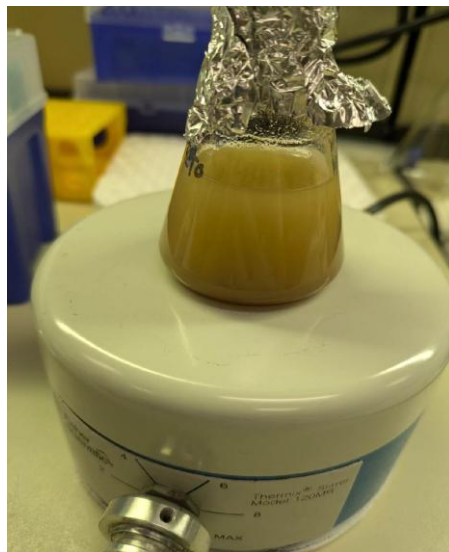

6.

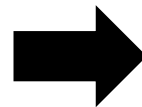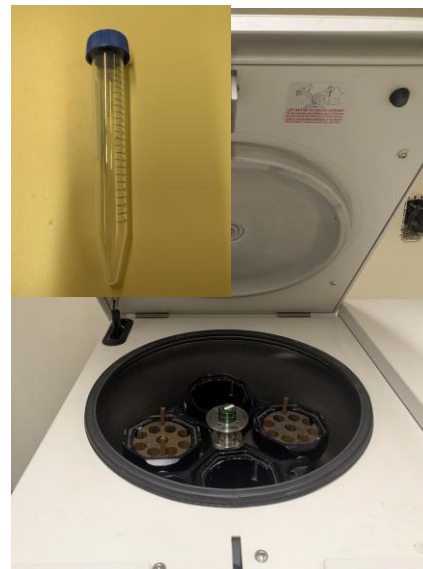

7.

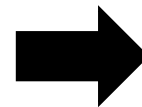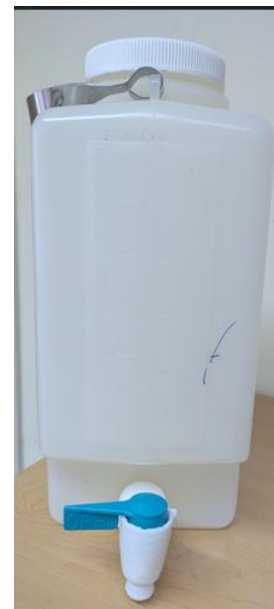

8.

Supplement: Supplementary file 1 [file mmc1.pdf]

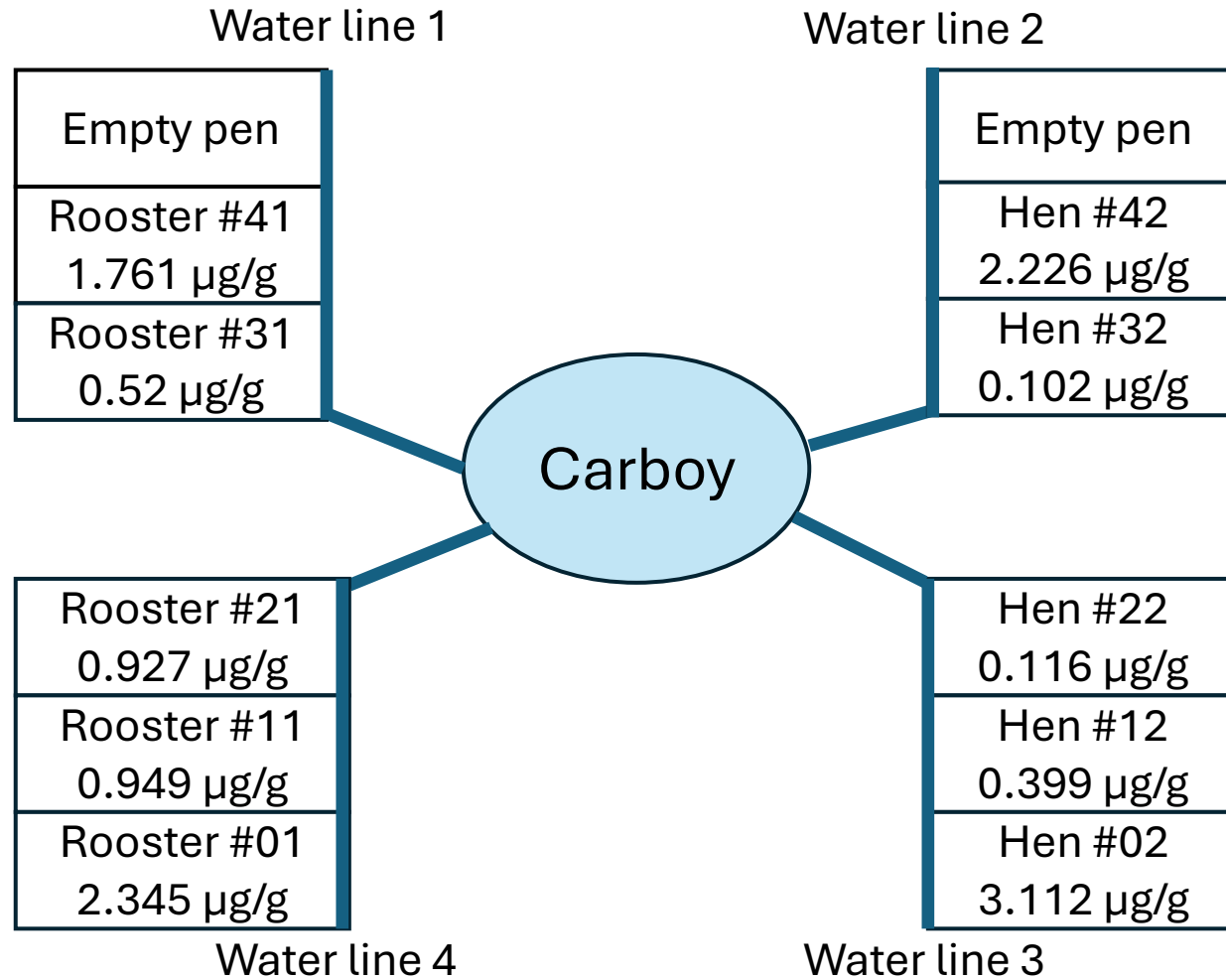

Supplement: Supplementary file 2 [file mmc2.pdf]
