# Supplementary material for: Large-bodied squab pigeons (Columba livia domestica) as a genetic treasure from Central Europe
Source: Poult Sci. 2025 Sep 28;104(12):105905. doi: 10.1016/j.psj.2025.105905 (PMC12549551; doi:10.1016/j.psj.2025.105905)
Supplement: Supplementary file 3 [file mmc3.pdf]

GIANT PIGEON: GENETIC TREASURE

Large-bodied squab pigeons (*Columba livia domestica*) as a genetic treasure from Central Europe

K. Balog[*, ‡], Sz. Kusza[*], Z. Bagi[*,1]

Table S2. Samples from GenBank used in this study

NCBI ID	Origin of the samples
>KC576929.1	Poland
>KC576928.1	Poland
>KC576927.1	Poland
>KC576926.1	Poland
>KC576925.1	Poland
>KC576924.1	Poland
>KC576923.1	Poland
>KC576922.1	Poland
>KC576921.1	Poland
>KC576920.1	Poland
>KC576919.1	Poland
>KC576918.1	Poland
>KC576917.1	Poland
>MK262652.1	New-Zealand
>MK262351.1	New-Zealand
>MG373562.1	Egypt
>MG271864.1	Egypt
>MG271863.1	Egypt
>JF498761.1	Irak
>JF498842.1	Hawaii, Kauai Island
>GU571831.1	Stockholm
>GU571830.1	Stockholm
>GU571343.1	Oslo
>GQ481606.1	Russia - Gorno-Altajszk
>GQ481605.1	Russia - Mongun-tajgai járás
>KU722397.1	East Africa, Djibouti
>AY666494.1	North America
>JN793567.1	India
>FJ808631.1	South Korea
>EF373367.1	Kanada
>DQ432860.1	Florida
>KP975238.1	India
>KP975237.1	India
>KP975216.1	India
>KC439307.1	India

[1] Correspondence should be addressed to Zoltán Bagi, Centre for Agricultural Genomics and Biotechnology, University of Debrecen, 4032, Debrecen, Hungary, Tel:
+36 52 508 444 / 88521, 68304, Email: bagiz@agr.unideb.hu

>KC182081.1	Pakistan
>KC182080.1	Pakistan
>KC182079.1	Pakistan
>KC182078.1	Pakistan
>KC182077.1	Pakistan
>KC182076.1	Pakistan
>KC182075.1	Pakistan
>KC182074.1	Pakistan
>KC182073.1	Pakistan
>KC182072.1	Pakistan
>JN850761.1	America
>JN850758.1	America
>JN850716.1	America
>FJ027421.1	Argentina, Buenos Aires
>AB753750.1	Saudi Arabia
