# Supplementary material for: Large-bodied squab pigeons (Columba livia domestica) as a genetic treasure from Central Europe
Source: Poult Sci. 2025 Sep 28;104(12):105905. doi: 10.1016/j.psj.2025.105905 (PMC12549551; doi:10.1016/j.psj.2025.105905)
Supplement: Supplementary file 4 [file mmc4.pdf]

GIANT PIGEON: GENETIC TREASURE

Large-bodied squab pigeons (*Columba livia domestica*) as a genetic treasure from Central Europe

K. Balog[*, ‡], Sz. Kusza[*], Z. Bagi[*,1]

Table S3. Summary of genetic diversity indices for 13 microsatellite loci

Loci	Marker	Observed Heterozygosity (H_O)	Expected Heterozygosity (H_E)	Fixation-index (F_{ST})	Inbreeding coefficient (F_{IS})	Inbreeding coefficient of an individual (I) relative to the total population (T) (F_{IT})	Geneflow (Nm)	Polymorphic information content (PIC)
L22	CliµT47	0.450	0.843	0.042	0.466	0.466	5.646	0.860
L21	PG2/CliMT24/ CliµT24	0.469	0.880	0.017	0.467	0.467	14.201	0.880
L01	CliµT17	0.566	0.890	0.053	0.364	0.364	4.467	0.934
L27	PG4	0.526	0.873	0.056	0.398	0.398	4.230	0.895
L28	PG5	0.208	0.746	0.035	0.721	0.721	6.913	0.764
n.d.	PG6	0.351	0.810	0.037	0.567	0.567	6.507	0.828
L30	PG7	0.406	0.870	0.056	0.533	0.533	4.230	0.913
L03	CliµD01	0.626	0.902	0.028	0.305	0.305	8.679	0.918
n.d.	CliµT13	0.399	0.795	0.054	0.498	0.498	4.414	0.823
L06	UU-Cli11	0.408	0.742	0.076	0.450	0.450	3.061	0.746
L25	CliµD16	0.406	0.803	0.028	0.495	0.495	8.743	0.773
L14	UU-Cli14	0.332	0.774	0.098	0.572	0.572	2.298	0.840
L24	CliµD35	0.568	0.858	0.054	0.338	0.338	4.405	0.897

[1] Correspondence should be addressed to Zoltán Bagi, Centre for Agricultural Genomics and Biotechnology, University of Debrecen, 4032, Debrecen, Hungary, Tel: +36 52 508 444 / 88521, 68304, Email: bagiz@agr.unideb.hu
