# Supplementary material for: Large-bodied squab pigeons (Columba livia domestica) as a genetic treasure from Central Europe
Source: Poult Sci. 2025 Sep 28;104(12):105905. doi: 10.1016/j.psj.2025.105905 (PMC12549551; doi:10.1016/j.psj.2025.105905)
Supplement: Supplementary file 5 [file mmc5.pdf]

GIANT PIGEON: GENETIC TREASURE

Large-bodied squab pigeons (*Columba livia domestica*) as a genetic treasure from Central Europe

K. Balog[*, ‡], Sz. Kusza[*], Z. Bagi[*,1]

Table S4. Summary of F_{IS} inbreeding coefficient in the level of breeds

Loci	Marker	Buga pigeon	Hungarian Cropper pigeon	Hungarian Giant pigeon	King	Mondain	Runt pigeon	Salonta Giant pigeon
L22	CliμT47	0.629	0.675	0.376	0.624	0.094	0.368	0.495
L21	PG2/CliMT24/ CliμT24	0.607	0.372	0.428	0.292	0.476	0.512	0.582
L01	CliμT17	0.188	0.511	0.354	0.410	0.333	0.479	0.288
L27	PG4	0.294	0.433	0.234	0.208	0.336	0.596	0.672
L28	PG5	0.504	0.740	0.928	1.000	0.424	0.811	0.700
n.d.	PG6	0.616	0.483	0.517	0.436	0.906	0.570	0.590
L30	PG7	0.603	0.384	0.369	0.488	0.599	0.516	0.778
L03	CliμD01	0.547	0.250	0.270	0.277	0.526	0.170	0.089
n.d.	CliμT13	0.555	0.382	0.362	0.900	0.480	0.427	0.469
L06	UU-Cli11	0.636	0.397	0.550	0.431	0.397	0.189	0.607
L25	CliμD16	0.608	0.762	0.298	0.333	0.590	0.465	0.414
L14	UU-Cli14	0.595	0.323	0.826	0.680	0.854	0.412	0.459
L24	CliμD35	0.267	0.129	0.210	0.439	0.548	0.247	0.571

[1] Correspondence should be addressed to Zoltán Bagi, Centre for Agricultural Genomics and Biotechnology, University of Debrecen, 4032, Debrecen, Hungary, Tel:
+36 52 508 444 / 88521, 68304, Email: bagiz@agr.unideb.hu
